# Computational Prediction of Subjective Human Immunodeficiency Virus Status in Malawi Using a Random Forest Approach

**DOI:** 10.1155/2019/5849183

**Published:** 2019-09-16

**Authors:** Sally Sonia Simmons

**Affiliations:** ^1^Institute of Demography, National Research University–Higher School of Economics, Myasnitskaya, 9/11, Moscow 101000, Russia; ^2^Department of Population and Health, University of Cape Coast, Cape Coast, Ghana

## Abstract

An individual's subjective judgment about his or her Human Immunodeficiency Virus status depends on certain factors, behavioral, health, and sociodemographic alike. This paper aims to develop a model with good accuracy for predicting subjective HIV infection status using the random forest approach. A total of 12,796 responses of Malawians over a 12-year period were assessed. Fourteen risk factors including behavioral, health, and sociodemographic information were analysed as potential predictors of subjective Human Immunodeficiency Virus infection status in the general population and thirteen behavioral, health, and sociodemographic information were analysed among males and females. The random forest approach was adopted to build a comprehensive model comprising 14 risk factors in Malawi. It was revealed that age, worries about infection, and health rate were the most significant predictors as compared to use of condoms, marital status, and education which were the least important predictors of subjective Human Immunodeficiency Virus status in Malawi. However, the importance of infidelity on the part of a spouse and marital status as predictors of subjective Human Immunodeficiency Virus status alternated among males and females. The importance of infidelity and marital status was relatively high among females than among males. The model achieved a prediction accuracy of about 97%–99% measured by c-statistic with jack-knife cross validation and verified by Mathews correlation coefficient. As a result, RF based model has great potential to be an effective approach for analysing subjective health status.

## 1. Introduction

Human Immunodeficiency Virus (HIV) infection is diagnosed based on antibody testing. Such a response to the HIV epidemic forms part of the primary prevention strategies in Malawi, where an estimated 11% of the population in 2012 have been infected [1]. However, the uptake of this test is influenced by individuals' thoughts about their status and further explained by certain factors; behavioral, health, and sociodemographic. That is to say that an individual's perception about his or her HIV status builds on gender, age, marital status, level of education, form of marriage, multiple sexual relationships, deteriorating health, and condom use [2–4]. Nevertheless, the impact of gender, age, level of education, form of marriage, multiple sexual relationships, marital status, deteriorating health, and contraception on the personal thoughts of Malawians HIV status have not to be computed appropriately.

In Malawi, multiple sexual partnerships (MSP) are a widespread norm in the young and mature adult years of life. At least two sexual partnerships occur in a year if a Malawian identifies with these age groups. On the one hand, MSP is prevalent among males than females, and on the other hand occurs regardless of marital status. Besides, within these form of relationships, certain social norms allow males to be unfaithful than females. In the youthful ages, for instance, about 16% to 2% of males and females have two or more sexual relationships in 12 months [4–6]. Possibly, these experiences of Malawians exacerbate their HIV status thoughts [7]. Educational attainment has been documented to have a dual influence on HIV in countries with a high prevalence rate. While higher infection rate has been associated with education, education might increase a person's subjective knowledge about his or her HIV status [2]. Further, it assists with the recognition of HIV infection signs and symptoms including respiratory infections, rashes weight loss, and other health failures [8]. Moreover, there is a high consensus that polygamy, a form of marital union, is endemic in sub-Saharan African countries like Malawi. Similarly, this behavior is accompanied by sexual acts without condoms [9]. A salient feature of this form of union is that it increases the density of a network of individuals and deflates the geodesics of infection transmission. Given this conviction, Malawians might have a subjective assessment of their HIV status [10].

Despite these postulations, most studies about HIV in Malawi measure access to HIV test, the people's perception of HIV, predictors of residence specific HIV rate, or whether a person has received a test or not [11, 12]. Only a handful of studies have undertaken a cross-sectional study that examines the subjective expectation of HIV in the country [6, 13]. There is, therefore, the need to understand and generalize the extent to which subjective HIV status in Malawi, where the onset of the HIV epidemic was earlier than most sub-Saharan African countries [14, 15], has transcended the 20th century to the 21st century.

## 2. Materials and Methods

### 2.1. Data and Data Source

The study used data from the Malawi Longitudinal Study of Families and Health (MLSFH) survey, documented from 1998 to 2010. MLSFH is a longitudinal cohort study investigating the varying degrees of HIV and other Sexually Transmitted Infection (STI) risks in the context of sub-Saharan Africa. Initially, the survey focused on the influence of social networks on fertility behavior and perception of HIV risk. Over time, the scope of the survey widened to include family/household dynamics, sexual behaviors, and intergenerational relations [6]. A detailed description of the MLSFH survey data and methods including, data collection, and quality are provided on the *project website* and in Kohler et al. (2015) work devoted to “*Cohort Profile: The Malawi Longitudinal Study of Families and Health (MLSFH)*.”

One thousand five hundred thirty-nine males (1,539) and 1,762 females were selected periodically to form the 12,769 participants sampled for the present study (see Table 1). A total of 15 features were selected for the present study. These became sex, expectation of infection, marital status, educational attainment, ever used condom, worried about infection, sexual partners in 12 months, infidelity, current health rate, comparison of current to previous health rate, number of wives, number of sexual partners, safer sex (condoms use with spouse), study period, and age. The selection of the features followed prior knowledge and achievement of a practically useful model [16]. The expectation of infection (outcome feature) was coded to become a binary variable (“no” = 1 or “yes”=0) instead of a polychotomous variable. Except for the outcome variable, missing values in all other variables were imputed using the predictive mean matching multiple imputation approach [17].

### 2.2. Statistical Analyses

The data for the study were divided into two separate categories; train and test in accordance with sex (male and female). For the general population of the study, the train data contained 8,973 entries (70%); the test data had 3,796 entries (30%). Among males, 3,019 and 1,292 were trained and tested. For females, 5,958 and 2,500 were the samples trained and tested. These data selection mechanisms worked to facilitate the convergence of the models, reduce overfitting and enhance the generalizability of the results (see Table 1) [18, 19].

The prediction method employed in the study was the random forest (RF) algorithm. It is one of the frequently used machine learning techniques in various prediction studies in the fields of sociology and biology. It is an ensemble learning approach for classifying several decision trees. Each tree is constructed using a bootstrap sample of the data. These samples comprise the training set for building the tree; that is, at each split the set of variables used becomes the random subset of the variables. This approach employs both bootstrap aggregation and random variable selection for constructing the trees. They help to produce unpruned trees, but each produces a vote suggesting one category or class. The RF then selects the class with the highest number of votes in comparison with other trees [20]. A detailed description RF algorithm can be found in J. Rogers and S. Gunn, [21]. These features of the RF approach reduces bias and variance among individual trees [19]. The importance of the predictors was assessed to understand the impact each predictor had on the outcome, subjective HIV status.

The performance of the model used in the study was evaluated using the jack-knife cross-validation. This cross-validation technique assessed the validity of the statistic. Here, the average of the subset of the data was computed and compared with the entire data to flag overfitting or selection bias in outcomes [19]. The performance model was derived from four scalar quantities: TP (True Positive HIV subjective status); FP (False Positive HIV subjective status); TN (True Negative HIV subjective status); FN (False Negative HIV subjective status). The accuracy of the measurement, the fraction of correctly specified expectations among all the predictions, was estimated as

(1) $$\begin{array}{c} \frac{\left(TP+TN\right)}{TP+FP+TN+FN}*100 \end{array}$$

The sensitivity of the measurement, corrected predicted HIV status became

(2) $$\begin{array}{c} \frac{\mathrm{TP}}{\left(TP+FN\right)}*100 \end{array}$$

The specificity of the measurement, corrected predicted none HIV status became

(3) $$\begin{array}{c} \frac{\mathrm{TN}}{\left(TN+FP\right)}*100 \end{array}$$

The prediction performance of the model used in the study was evaluated by the Mathews Correlation Coefficient (MCC) [19]. It was estimated as:

(4) $$\begin{array}{c} \frac{TP*TN-FP*FN}{\sqrt{\left(TP+TN\right)\left(TP+FN\right)\left(FP+TN\right)\left(FP+FN\right)}}*100 \end{array}$$

A receiver operating characteristic (ROC) curve was generated to display the outcome of the measurement produced from the binary classification. The curve assisted with the generation of the c-statistic (area under the curve [AUC]). The AUC was used to evaluate the potency of discriminating the true subjective HIV status knowledge among Malawians [22]. All analyses and simulations were carried out with *R Programme*.

## 3. Results

**Figure 1:**
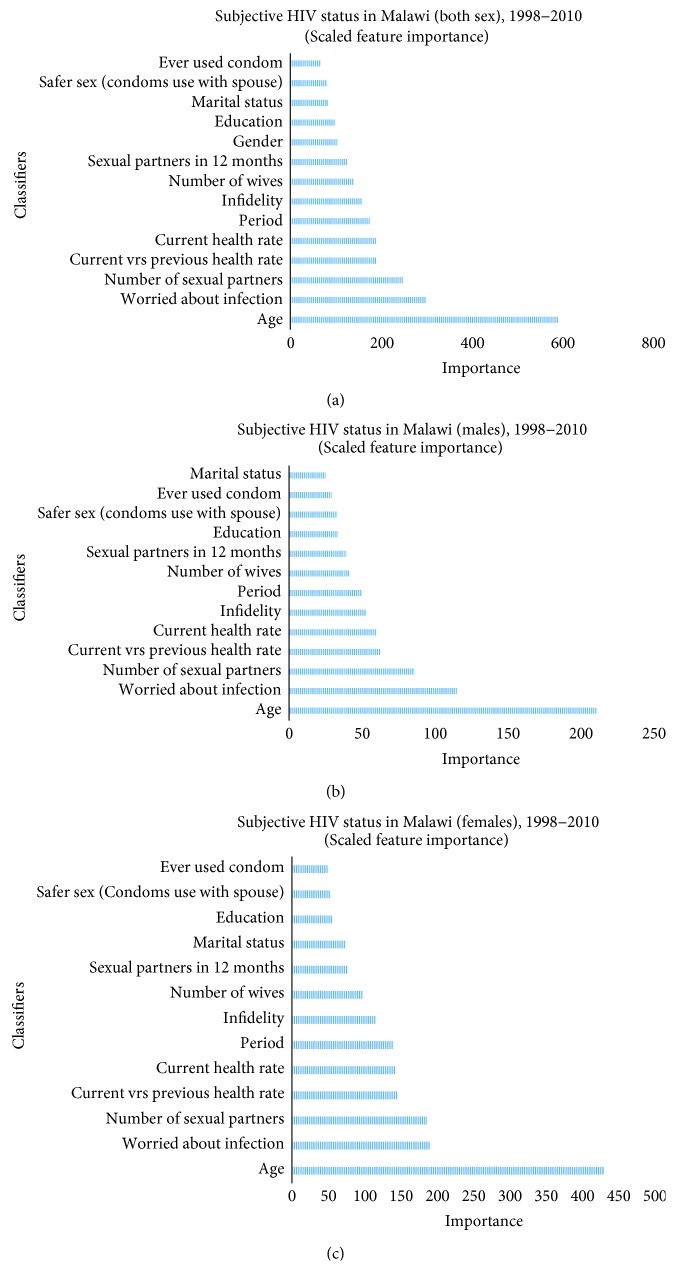
Importance of classifiers.

Figure 1 shows the importance of classifiers for predicting subjective HIV status among Malawians. Generally, the figure shows that age is the most critical determining factor for predicting Malawians subjective HIV status. It is followed by the total number of sexual partners of a Malawian, how worried a Malawian is about his or her HIV status and health rate. On the contrary, ever use of condom was the weakest determinant for predicting subjective HIV status in Malawi. It was preceded by the marital status of a Malawian and preference for safer sex. Age, worries about infection, and the number of sexual partners were the main predictors influencing personal HIV status thoughts among Malawian males. The personal HIV status knowledge among males was less predicted by marital status, use of condom, safer sex with spouse, and education although marital status was less important in predicting the event. Like males, age, worries about infection, and the number of sexual partners were the main predictors influencing personal HIV status thoughts among Malawian women. However, the ever use of condom was the least important determinant of subjective HIV status knowledge. Instead, it was followed by safer sex, education, marital status, and sexual partners in the last 12 months. While infidelity ranked sixth as an essential determinant for predicting subjective HIV status knowledge among females, it was fifth among males.

Table 2 shows the output of the confusion matrix employed in the study. Generally, both sex trained data achieved 97.24% accuracy. It was complemented by a sensitivity value of 86.59%, specificity value of 99.94%, and MCC of 76.47%. The accuracy, sensitivity, specificity, and MCC from the tested data were 98.63%, 93.79%, 99.97%, and 74.83%, respectively. However, these varied among males and females. Males, unlike females, achieved higher accuracy with the training data than the testing data. Also, males, unlike females, had sensitivity with the test data than training data. MCC value for females was higher for the test data.

**Figure 2:**
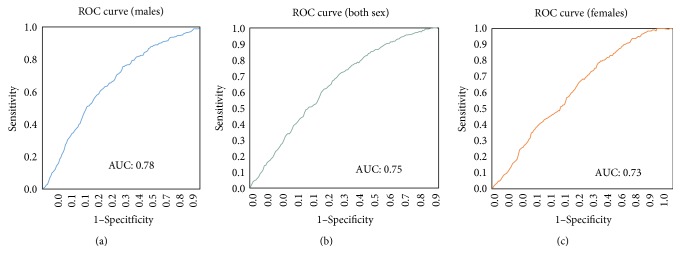
A ROC curve for subjective HIV status true positive and false positive rate.

Figure 2 shows the ROC curve and the area under the curve (AUC) for the three populations under study, males, females, and both sex. The predictive ability of the classifiers (AUC) was about 73%–78% and denoted an acceptable to excellent discrimination outcome. While males had the highest c-statistic (78%), females observed the lowest (73%). From the figure, all points are above the *correctly classified positive subjective HIV status=incorrectly classified negative subjective HIV status threshold*.

## 4. Discussion

The present study assessed the importance of factors determining the subjective HIV status declaration among Malawians. It identified participant characteristics that predicted a Malawian's subjective judgment about his or her HIV status. Generally, a Malawian had a subjective knowledge about his or her HIV status depending on age, how worried the person is about the infection, health status, multiple partners, or infidelity than if the person ever used a condom, preferred protected sex with a spouse, or is educated. Age predicted about 24.56% of all personal HIV status knowledge all the participants. This may be a sign of multiple sexual partnership experiences in the past or in present times as A. Delavande and H.-P. Kohler [6] and N. Wilson Chialepeh and A. Sathiyasusuman, [4] indicated that the youthful ages through to the mature adult life years of Malawians is characterised by multiple sexual partnerships. Such partnerships, according to Steffenson et al. [7] increase the spread of infections transmitted sexually. The link between these factors and subjective HIV status could be opened to speculation. It could be that this concept influences thoughts and therefore determines people's knowledge about their HIV status. The prevailing doctrine of higher prevalence of HIV in Malawi before its rise in countries such as South Africa and Lesotho might also be a reason for the importance of age in predicting subjective HIV status. Persons born in the era of the high prevalence of HIV in Malawi may consider themselves to be infected with HIV as compared to others since the latter group might be young and mature adults as at the period [14, 15].

Between males and females, the main distinguishing features of importance were infidelity and marital status. While infidelity of spouse predicted approximately 6.43% of all personal thoughts of HIV among males, it was about 6.97% among females. The variance in the importance of infidelity of spouse reflects the fact that although the risk of contracting the infection from a spouse is likely, females are more like to be infected with HIV from their husbands than husbands contracting the disease from his spouse. Thus, females subject themselves to be infected with HIV due to husband's unfaithfulness than males think they are infected with HIV because of wife's unfaithfulness [5].

In this study, subjective HIV status in Malawi was predicted using the random forest (RF) approach. This machine learning approach was used to learn the patterns in longitudinal data points to further predict personal HIV knowledge in Malawi in the general and among males and females in the country. The high specificity as compared to the sensitivity reflects the accurate detection of the Malawians who do not subjectively perceive themselves as being infected with HIV. The lower values of the sensitivity as compared to the specificity indicate the inability to classify all Malawians who perceive themselves to be infected with HIV. The AUC above further illustrates the fact that the proportion of correctly classified Malawians who identified with a positive subjective HIV status is higher than the proportion of incorrectly classified Malawians who did not identify with a positive subjective HIV status.

## 5. Conclusions

In this paper, a model that combines sociodemographic and behavioral risk factors for predicting the subjective HIV status in Malawi using random forest machine learning approach was developed. The RF model showed the importance of features employed in the study as predictors of subjective HIV status knowledge. It showed the separation of positive subjective HIV status from negative subjective HIV status using a jack-knife cross-validation, MCC, and ROC methods. Generally, age was the most significant predictor of personal HIV infection status in contrast to marital status or ever used a condom, which were the least import determinants. Out of every 100 Malawians, the RF approach was able to predict about 86–99 people's subjective HIV infection status. Therefore, RF has been demonstrated to be a potentially useful approach for the analysis of subjective health state.

The omission of some potential predictors, such as hospital visits or ever tested for HIV in the model adopted by this study is a limitation. It was due to incomplete data and the possible effects of the validity of the results of included. Thus, a comprehensive model can be developed if these potential predictors were added to generate better discrimination performance.

## Figures and Tables

**Table 1:** Training and testing data used in the study.

Data	Category	Subjective HIV status
Training data	Both sex	8,973
Testing data		3,796
Total		12,769
Training data	Males	3,019
Testing data		1,292
Total		4,311
Training data	Females	5,958
Testing data		2,500
Total		8458

*Source.* MLSFH, 1998–2010.

**Table 2:** Prediction results of the random forest method (confusion matrix) in the present study.

Data	Categories	Sensitivity (%)	Specificity (%)	Accuracy (%)	MCC (%)
Training	Both Sex	86.59	99.94	97.24	76.47
Testing		93.79	99.97	98.63	74.83
Training	Males	88.96	100.00	97.81	78.06
Testing		95.72	99.71	98.92	69.39
Training	Females	89.78	99.87	97.73	75.11
Testing		94.70	100.00	98.96	73.02

*Source.* MLSFH, 1998–2010. *Note.* All outputs were significant at *p* < 0.05.

## Data Availability

The data used to support the findings of this study are available from the corresponding author upon request.

## Abbreviations

HIV: Human immunodeficiency virus
MSP: Multiple sexual partnerships
MLSFH: Malawi longitudinal study of families and health
RF: Random forest
MCC: Mathews correlation coefficient
ROC: Receiver operating characteristic
AUC: Area under the curve (C-statistic).
